# (±)-2-{3-[1-(2,4-Difluoro­phen­yl)eth­yl]-1,3-thia­zolidin-2-yl­idene}malononitrile

**DOI:** 10.1107/S1600536811023737

**Published:** 2011-06-22

**Authors:** Lei Liu, Tao Song, Liang-zhong Xu

**Affiliations:** a College of Chemistry and Molecular Engineering, Qingdao University of Science and Technology, Qingdao 266042, People’s Republic of China

## Abstract

In the title compound, C_14_H_11_F_2_N_3_S, the heterocyclic five-membered ring has an envelope conformation. Although the mol­ecule is chiral, the compound is a racemate (*R*/*S*). There is a weak inter­molecular C—H⋯π inter­action but no classical hydrogen bonds are observed in the crystal structure.

## Related literature

For the biological activity of thia­zoles and thia­zolidines, see: Melnikov *et al.* (1979 ▶); Kratt *et al.* (1986 ▶). For the synthesis, see: Hense *et al.* (2002 ▶). For a related structure, see: Xu *et al.* (2005 ▶). For puckering parameters, see: Cremer & Pople (1975 ▶).
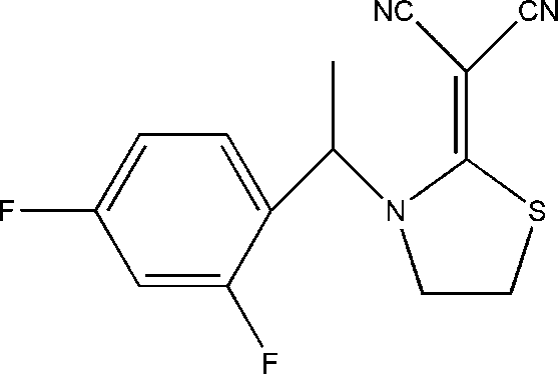


## Experimental

### 

#### Crystal data

C_14_H_11_F_2_N_3_S
*M* *_r_* = 291.32
Triclinic, 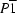

*a* = 7.6886 (14) Å
*b* = 8.9854 (16) Å
*c* = 10.8188 (19) Å
α = 102.508 (2)°
β = 90.940 (2)°
γ = 112.861 (2)°
*V* = 668.1 (2) Å^3^
*Z* = 2
Mo *K*α radiation
μ = 0.26 mm^−1^
*T* = 296 K
0.32 × 0.30 × 0.28 mm

#### Data collection

Bruker SMART CCD area-detector diffractometer
Absorption correction: multi-scan (*SADABS*; Sheldrick, 1996 ▶) *T*_min_ = 0.922, *T*_max_ = 0.931
4810 measured reflections
2332 independent reflections
2049 reflections with *I* > 2σ(*I*)
*R*_int_ = 0.031

#### Refinement

*R*[*F* ^2^ > 2σ(*F* ^2^)] = 0.041
*wR*(*F* ^2^) = 0.116
*S* = 1.08
2338 reflections
182 parameters
H-atom parameters constrained
Δρ_max_ = 0.27 e Å^−3^
Δρ_min_ = −0.31 e Å^−3^

### 

Data collection: *SMART* (Bruker, 1998 ▶); cell refinement: *SAINT* (Bruker, 1999 ▶); data reduction: *SAINT*; program(s) used to solve structure: *SHELXS97* (Sheldrick, 2008 ▶); program(s) used to refine structure: *SHELXL97* (Sheldrick, 2008 ▶); molecular graphics: *ORTEPIII* (Burnett & Johnson, 1996 ▶) and *ORTEP-3 for Windows* (Farrugia, 1997 ▶); software used to prepare material for publication: *SHELXL97*.

## Supplementary Material

Crystal structure: contains datablock(s) I, global. DOI: 10.1107/S1600536811023737/dn2699sup1.cif

Structure factors: contains datablock(s) I. DOI: 10.1107/S1600536811023737/dn2699Isup2.hkl

Supplementary material file. DOI: 10.1107/S1600536811023737/dn2699Isup3.cml

Additional supplementary materials:  crystallographic information; 3D view; checkCIF report

## Figures and Tables

**Table 1:** Hydrogen-bond geometry (Å, °) *Cg* is the centroid of the benzene ring.

*D*—H⋯*A*	*D*—H	H⋯*A*	*D*⋯*A*	*D*—H⋯*A*
C12—H12*B*⋯*Cg*1^i^	0.96	2.94	3.848 (3)	158

Symmetry code: (i) 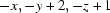.

**Fig. 1.**
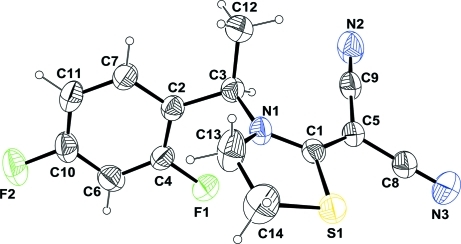
View of the title compound (I), with the atom labeling scheme. Displacement ellipsoids are drawn at the 50% probability level. H atoms are represented as small spheres of arbitrary radii.

## supplementary crystallographic information

### Comment

It has been reported that both thiazoles and thiazolidines have good and wide insecticidal, fungicidal, herbicidal and acaricidal activities (Melnikov, *et al.*, 1979; Kratt *et al.*, 1986). As part of our search for compounds with good herbicidal and fungicidal activity, the title compound, (I), was synthesized.

In (I) The heterocyclic five-membered ring (C1/C13/N1/C14/S1) has an envelope conformation on C13 with puckering parameters, Q(2)= 0.155 (3)Å and φ(2) = 108.1 (9)° (Cremer & Pople, 1975) (Fig. 1). The bond lengths and angles are within expected values for the thiazolidin ring (Xu, *et al.*, 2005). The phenyl ring is twisted with respect to the thiazolidin ring with a torsion angle of 138.0 (2)°. No classical hydrogen bonds were found in the crystal, only van der Waals forces and a weak C-H···π interaction involving the Cg1 centroid of a symetry related phenyl ring (Table 1) stabilize the crystal structure.

### Experimental

2-(thiazolidin-2-ylidene)malononitrile 15.5 g (0.1 mol), potassium carbonate 13.8 g (0.1 mol) and acetonitrile 50 g are charged in a flask equipped with stirrer and reflux condenser. The mixture is heated to reflux, then 1-(1-chloroethyl)-2,4-difluorobenzene 17.7 g (0.1 mol) is droped in over 30 minutes. Keep refluxing for 12 h. Upon cooling at room temperature. The reaction mixture is filtered, and the solution is concentrated under reduced pressure to give the title compound (I) 27.2 g (92% yield). (Hense, *et al.*, 2002). Single crystals suitable for X-ray measurement were obtained by recrystallization from the tetrahydrofuran solution of (I) at room temperature.

### Refinement

All C-bound H atoms were placed in calculated positions, with C—H = 0.95–1.00 Å, and included in the final cycles of refinement using a riding model, with *U*_iso_(H) = 1.2*U*_eq_(C) for the aryl and methylene H atoms and 1.5*U*_eq_(C) for methyl H atoms.

### Crystal data

**Table:**

C_14_H_11_F_2_N_3_S	*Z* = 2
*M**_r_* = 291.32	*F*(000) = 300
Triclinic, *P*1	*D*_x_ = 1.448 Mg m^−^^3^
Hall symbol: -P 1	Mo *K*α radiation, λ = 0.71073 Å
*a* = 7.6886 (14) Å	Cell parameters from 2422 reflections
*b* = 8.9854 (16) Å	θ = 2.3–25.1°
*c* = 10.8188 (19) Å	µ = 0.26 mm^−^^1^
α = 102.508 (2)°	*T* = 296 K
β = 90.940 (2)°	Block, colorless
γ = 112.861 (2)°	0.32 × 0.30 × 0.28 mm
*V* = 668.1 (2) Å^3^	

### Data collection

**Table:**

Bruker SMART CCD area-detector diffractometer	2332 independent reflections
Radiation source: fine-focus sealed tube	2049 reflections with *I* > 2σ(*I*)
graphite	*R*_int_ = 0.031
φ and ω scans	θ_max_ = 25.0°, θ_min_ = 1.9°
Absorption correction: multi-scan (*SADABS*; Sheldrick, 1996)	*h* = −9→9
*T*_min_ = 0.922, *T*_max_ = 0.931	*k* = −10→10
4810 measured reflections	*l* = −12→12

### Refinement

**Table:**

Refinement on *F*^2^	Primary atom site location: structure-invariant direct methods
Least-squares matrix: full	Secondary atom site location: difference Fourier map
*R*[*F*^2^ > 2σ(*F*^2^)] = 0.041	Hydrogen site location: inferred from neighbouring sites
*wR*(*F*^2^) = 0.116	H-atom parameters constrained
*S* = 1.08	*w* = 1/[σ^2^(*F*_o_^2^) + (0.0521*P*)^2^ + 0.2818*P*] where *P* = (*F*_o_^2^ + 2*F*_c_^2^)/3
2338 reflections	(Δ/σ)_max_ < 0.001
182 parameters	Δρ_max_ = 0.27 e Å^−^^3^
0 restraints	Δρ_min_ = −0.31 e Å^−^^3^

### Special details

**Table:**

Geometry. All esds (except the esd in the dihedral angle between two l.s. planes) are estimated using the full covariance matrix. The cell esds are taken into account individually in the estimation of esds in distances, angles and torsion angles; correlations between esds in cell parameters are only used when they are defined by crystal symmetry. An approximate (isotropic) treatment of cell esds is used for estimating esds involving l.s. planes.
Refinement. Refinement of *F*^2^ against ALL reflections. The weighted *R*-factor *wR* and goodness of fit *S* are based on *F*^2^, conventional *R*-factors *R* are based on *F*, with *F* set to zero for negative *F*^2^. The threshold expression of *F*^2^ > σ(*F*^2^) is used only for calculating *R*-factors(gt) *etc*. and is not relevant to the choice of reflections for refinement. *R*-factors based on *F*^2^ are statistically about twice as large as those based on *F*, and *R*- factors based on ALL data will be even larger.

### Fractional atomic coordinates and isotropic or equivalent isotropic displacement parameters (Å^2^)

**Table:**

	*x*	*y*	*z*	*U*_iso_*/*U*_eq_	
S1	0.28619 (9)	0.70036 (7)	−0.03503 (5)	0.0493 (2)	
F1	−0.19221 (18)	0.68270 (16)	0.17886 (11)	0.0523 (3)	
F2	−0.3465 (2)	0.44311 (19)	0.52735 (15)	0.0678 (4)	
C1	0.2408 (3)	0.8531 (2)	0.07280 (18)	0.0345 (4)	
C2	0.0263 (3)	0.7988 (2)	0.36404 (17)	0.0357 (4)	
N1	0.2234 (2)	0.8236 (2)	0.18869 (15)	0.0364 (4)	
C3	0.1633 (3)	0.9200 (2)	0.29655 (18)	0.0361 (4)	
H3	0.0944	0.9755	0.2609	0.043*	
C4	−0.1470 (3)	0.6816 (2)	0.30078 (18)	0.0380 (4)	
C5	0.2315 (3)	0.9856 (2)	0.03016 (18)	0.0366 (4)	
C6	−0.2757 (3)	0.5620 (3)	0.3520 (2)	0.0463 (5)	
H6	−0.3904	0.4852	0.3063	0.056*	
C7	0.0653 (3)	0.7939 (3)	0.48932 (19)	0.0419 (5)	
H7	0.1785	0.8718	0.5365	0.050*	
C8	0.2460 (3)	0.9825 (3)	−0.1015 (2)	0.0439 (5)	
C9	0.2356 (3)	1.1362 (3)	0.10832 (19)	0.0409 (5)	
N2	0.2449 (3)	1.2619 (2)	0.1680 (2)	0.0598 (6)	
C10	−0.2252 (3)	0.5624 (3)	0.4748 (2)	0.0465 (5)	
C11	−0.0604 (3)	0.6760 (3)	0.5452 (2)	0.0476 (5)	
H11	−0.0328	0.6746	0.6288	0.057*	
C12	0.3374 (3)	1.0542 (3)	0.3821 (2)	0.0504 (5)	
H12A	0.4102	1.0031	0.4156	0.076*	
H12B	0.2974	1.1161	0.4512	0.076*	
H12C	0.4140	1.1281	0.3335	0.076*	
N3	0.2605 (3)	0.9831 (3)	−0.20641 (19)	0.0652 (6)	
C13	0.2830 (4)	0.6928 (3)	0.2078 (2)	0.0634 (7)	
H13A	0.4078	0.7428	0.2559	0.076*	
H13B	0.1944	0.6238	0.2558	0.076*	
C14	0.2883 (5)	0.5919 (4)	0.0852 (3)	0.0751 (8)	
H14A	0.1790	0.4861	0.0671	0.090*	
H14B	0.4023	0.5701	0.0861	0.090*	

### Atomic displacement parameters (Å^2^)

**Table:**

	*U*^11^	*U*^22^	*U*^33^	*U*^12^	*U*^13^	*U*^23^
S1	0.0637 (4)	0.0392 (3)	0.0472 (3)	0.0227 (3)	0.0196 (3)	0.0102 (2)
F1	0.0570 (8)	0.0552 (8)	0.0364 (6)	0.0142 (6)	−0.0043 (5)	0.0111 (6)
F2	0.0685 (9)	0.0637 (9)	0.0746 (10)	0.0176 (7)	0.0297 (7)	0.0388 (8)
C1	0.0313 (9)	0.0328 (10)	0.0370 (10)	0.0098 (8)	0.0066 (8)	0.0092 (8)
C2	0.0404 (10)	0.0367 (10)	0.0332 (10)	0.0181 (9)	0.0077 (8)	0.0097 (8)
N1	0.0428 (9)	0.0359 (9)	0.0375 (9)	0.0193 (7)	0.0116 (7)	0.0158 (7)
C3	0.0417 (11)	0.0346 (10)	0.0340 (10)	0.0163 (8)	0.0056 (8)	0.0101 (8)
C4	0.0446 (11)	0.0404 (11)	0.0309 (9)	0.0195 (9)	0.0052 (8)	0.0075 (8)
C5	0.0398 (10)	0.0346 (10)	0.0347 (10)	0.0121 (8)	0.0055 (8)	0.0124 (8)
C6	0.0435 (11)	0.0400 (11)	0.0509 (12)	0.0129 (9)	0.0093 (9)	0.0089 (9)
C7	0.0447 (11)	0.0467 (12)	0.0337 (10)	0.0174 (9)	0.0045 (8)	0.0104 (9)
C8	0.0458 (12)	0.0423 (11)	0.0413 (12)	0.0132 (9)	0.0045 (9)	0.0142 (9)
C9	0.0464 (11)	0.0390 (11)	0.0422 (11)	0.0167 (9)	0.0106 (9)	0.0205 (9)
N2	0.0860 (15)	0.0423 (11)	0.0621 (13)	0.0318 (11)	0.0249 (11)	0.0224 (10)
C10	0.0529 (13)	0.0453 (12)	0.0490 (12)	0.0220 (10)	0.0229 (10)	0.0212 (10)
C11	0.0592 (14)	0.0574 (13)	0.0353 (10)	0.0283 (11)	0.0140 (10)	0.0192 (10)
C12	0.0515 (13)	0.0445 (12)	0.0461 (12)	0.0094 (10)	−0.0007 (10)	0.0120 (10)
N3	0.0812 (15)	0.0721 (15)	0.0408 (11)	0.0256 (12)	0.0064 (10)	0.0205 (10)
C13	0.096 (2)	0.0681 (16)	0.0625 (15)	0.0598 (16)	0.0324 (14)	0.0371 (13)
C14	0.124 (3)	0.0629 (16)	0.0657 (17)	0.0628 (18)	0.0195 (16)	0.0223 (13)

### Geometric parameters (Å, °)

**Table:**

S1—C1	1.7490 (19)	C6—C10	1.376 (3)
S1—C14	1.789 (3)	C6—H6	0.9300
F1—C4	1.362 (2)	C7—C11	1.387 (3)
F2—C10	1.359 (2)	C7—H7	0.9300
C1—N1	1.335 (2)	C8—N3	1.144 (3)
C1—C5	1.392 (3)	C9—N2	1.148 (3)
C2—C4	1.386 (3)	C10—C11	1.362 (3)
C2—C7	1.397 (3)	C11—H11	0.9300
C2—C3	1.519 (3)	C12—H12A	0.9600
N1—C13	1.468 (3)	C12—H12B	0.9600
N1—C3	1.488 (2)	C12—H12C	0.9600
C3—C12	1.524 (3)	C13—C14	1.447 (4)
C3—H3	0.9800	C13—H13A	0.9700
C4—C6	1.375 (3)	C13—H13B	0.9700
C5—C9	1.421 (3)	C14—H14A	0.9700
C5—C8	1.425 (3)	C14—H14B	0.9700
			
C1—S1—C14	91.83 (11)	C2—C7—H7	119.1
N1—C1—C5	129.40 (18)	N3—C8—C5	178.6 (2)
N1—C1—S1	112.28 (14)	N2—C9—C5	176.8 (2)
C5—C1—S1	118.31 (14)	F2—C10—C11	118.9 (2)
C4—C2—C7	115.89 (18)	F2—C10—C6	118.0 (2)
C4—C2—C3	120.40 (17)	C11—C10—C6	123.1 (2)
C7—C2—C3	123.69 (18)	C10—C11—C7	118.5 (2)
C1—N1—C13	114.67 (16)	C10—C11—H11	120.8
C1—N1—C3	124.89 (15)	C7—C11—H11	120.8
C13—N1—C3	120.28 (16)	C3—C12—H12A	109.5
N1—C3—C2	108.53 (15)	C3—C12—H12B	109.5
N1—C3—C12	110.00 (17)	H12A—C12—H12B	109.5
C2—C3—C12	114.49 (16)	C3—C12—H12C	109.5
N1—C3—H3	107.9	H12A—C12—H12C	109.5
C2—C3—H3	107.9	H12B—C12—H12C	109.5
C12—C3—H3	107.9	C14—C13—N1	109.3 (2)
F1—C4—C6	117.54 (18)	C14—C13—H13A	109.8
F1—C4—C2	118.02 (17)	N1—C13—H13A	109.8
C6—C4—C2	124.43 (19)	C14—C13—H13B	109.8
C1—C5—C9	125.92 (17)	N1—C13—H13B	109.8
C1—C5—C8	117.88 (18)	H13A—C13—H13B	108.3
C9—C5—C8	115.58 (17)	C13—C14—S1	109.04 (17)
C4—C6—C10	116.3 (2)	C13—C14—H14A	109.9
C4—C6—H6	121.8	S1—C14—H14A	109.9
C10—C6—H6	121.8	C13—C14—H14B	109.9
C11—C7—C2	121.7 (2)	S1—C14—H14B	109.9
C11—C7—H7	119.1	H14A—C14—H14B	108.3

### Hydrogen-bond geometry (Å, °)

**Table:**

Cg is the centroid of the phenyl ring.

**Table:**

*D*—H···*A*	*D*—H	H···*A*	*D*···*A*	*D*—H···*A*
C12—H12B···Cg1^i^	0.96	2.94	3.848 (3)	158

Symmetry codes: (i) −*x*, −*y*+2, −*z*+1.
